# Synthesis, characterization, and in vitro–in ovo toxicological screening of silibinin fatty acids conjugates as prodrugs with potential biomedical applications

**DOI:** 10.17305/bb.2024.10600

**Published:** 2024-12-01

**Authors:** Cristina Dehelean, Ersilia Alexa, Iasmina Marcovici, Andrada Iftode, Geza Lazar, Andrea Simion, Vasile Chis, Adrian Pirnau, Simona Cinta Pinzaru, Estera Boeriu

**Affiliations:** 1 Faculty of Pharmacy, “Victor Babes” University of Medicine and Pharmacy Timisoara, Timisoara, Romania; 2 Research Center for Pharmaco-Toxicological Evaluations, Faculty of Pharmacy, “Victor Babes” University of Medicine and Pharmacy Timisoara, Timisoara, Romania; 3 Faculty of Food Engineering, University of Life Sciences “King Michael I” from Timisoara, Timisoara, Romania; 4 “Ioan Ursu” Institute of the Faculty of Physics, Babes-Bolyai University, Cluj-Napoca, Romania; 5 RDI Institute of Applied Natural Sciences, Babeş-Bolyai University, Cluj-Napoca, Romania; 6 National Institute of Research and Development of Isotopic and Molecular Technologies, Cluj-Napoca, Romania; 7 Faculty of Medicine, Department of Pediatrics, “Victor Babes” University of Medicine and Pharmacy Timisoara, Timisoara, Romania; 8 Department of Oncology and Hematology, “Louis Turcanu” Emergency Clinical Hospital for Children, Timisoara, Romania

**Keywords:** Silibinin (SIL), oleic acid (OA), linoleic acid (LA), derivatization, physicochemical characterization, cytotoxicity, cell migration, irritant effect

## Abstract

Silibinin (SIL), the most active phytocompound from *Silybum marianum* (L.), exerts many biological effects but has low stability and bioavailability. To overcome these drawbacks, the current research proposed the synthesis of silibilin oleate (SIL-O) and silibilin linoleate (SIL-L) derivatives as prodrugs with potentially optimized properties for biomedical applications, and the establishment of their in vitro–in ovo safety profiles. The physicochemical characterization of the obtained compounds using density functional theory (DFT) calculations, and Raman and ^1^H liquid-state nuclear magnetic resonance (NMR) spectroscopy confirmed the formation of SIL-O and SIL-L complexes. Computational predictions revealed that these lipophilic derivatives present a lower drug-likeness score (−29.96 for SIL-O and −23.55 for SIL-L) compared to SIL, but an overall positive drug score (0.07) and no risk for severe adverse effects. SIL-O and SIL-L showed no cytotoxicity or impairment in cell migration at low concentrations, but at the highest concentration (100 µM), they displayed distinct toxicological profiles. SIL-L was more cytotoxic (on cardiomyoblasts – H9c2(2-1), hepatocytes – HepaRG, and keratinocytes– HaCaT) than SIL-O or SIL, significantly inhibiting cell viability (< 60%), altering cellular morphology, reducing cell confluence (< 70%), and inducing prominent apoptotic-like nuclear features. At the concentration of 100 µM, SIL-O presented an irritation score (IS) of 0.61, indicating a lack of irritant effect on the chorioallantoic membrane (CAM), while SIL-L was classified as a slight irritant with an IS of 1.99. These findings outline a more favorable in vitro and in ovo biocompatibility for SIL-O compared to SIL-L, whose applications are dosage limited due to potential toxicity.

## Introduction

The popularity of medicinal herbs has increased globally over the past years, becoming of utmost importance in the treatment of various medical settings. The majority of today’s medicines owe their origin to medicinal plants [1] which still serve as an unexhausted resource for bioactive compounds with potent biological activities and applications in different therapeutic areas [2, 3]. Medicinal plants and their bioactive phytochemicals present the advantage of minimal side effects and high pharmaceutical value to combat various diseases in humans [4–6].

One such medicinal plant is *Silybum marianum* L. Gaertn. (also named milk thistle) which is mainly recommended as a natural remedy in the treatment of liver or gallbladder dysfunctions despite its broad spectrum of other therapeutic activities (e.g., anti-inflammatory, antioxidant, antineoplastic, cardio-, and neuro-protective, etc.) [7–9]. The biologically active extract from *S. marianum* L., known as silymarin, protects the plant from both biotic and abiotic stressors (e.g., bacteria, fungi, and radiation), being a mixture of four phytocompounds – silibinin (or silibin), silychristin, silydianin, and isosilybin. Silibinin (SIL), the most abundant and therapeutically active compound existing in the silymarin complex [7, 10], is a polyphenolic antioxidant with a flavolignan structure formed of two diastereomers – SIL A and B [7, 11], and a compound with increased safety, associated only with minor adverse effects at the gastrointestinal level [12]. In terms of therapeutic applications, SIL has been historically used for its hepatoprotective properties, being officially approved for the treatment of liver diseases since the 1970s [7]. More recently, SIL was associated with protective effects against chemically induced cardiotoxicity and showed a potential utility in the treatment of heart disorders such as myocardial ischemia [13, 14]. Additionally, SIL presents skin health-improving properties, by reducing the risk of cutaneous cancers and conferring protection against chemical oxidants or ultraviolet radiation-induced damage [15–17]. However, despite being included as an active ingredient in conventional formulations such as tablets and capsules which are administered orally, SIL (along with other silymarin constituents) suffers from limited intestinal resorption and oral bioavailability, mainly due to its poor solubility in water and lipid media [18, 19]. Pharmacokinetic studies revealed that its absorption from the gastrointestinal tract upon oral administration is about 23%–47%. SIL also suffers an intense phase II metabolization, being rapidly excreted in bile and urine which significantly lowers its therapeutic efficiency [12, 18]. Furthermore, pure SIL was found to be unstable in buffers possessing a pH of 1.0–7.8, as well as physiological media (i.e., plasma, intestinal, and liver fluids) [20].

Prodrugs, which are defined as inactive drug conjugates that efficiently reconstitute the parent compound following in vivo administration, are extensively investigated as an efficient approach to optimize the inadequate pharmacokinetic and pharmacodynamic features of bioactive compounds, such as poor solubility, reduced chemical stability, limited bioavailability, extensive metabolization, scarce penetration through biological barriers, and low site-specificity or therapeutic efficiency. The conventional prodrug strategy resorts to drug derivatization using either hydrophilic or lipophilic functional groups [21]. Lipidic molecules have been broadly employed as building blocks for the obtainment of various drug precursors, their attachment to active agents improving drug lipophilicity, retention, pharmacokinetic profile, and therapeutic activity, while also facilitating their incorporation in advanced delivery systems [22, 23]. Lipidic derivatization was previously employed for SIL, its conjugation with phosphatidylcholine resulting in the obtainment of a complex (Silipide) with enhanced bioavailability and antioxidant activity [24].

Fatty acids (FAs) are lipid-based compounds well known for their structural, energetic, and nutritional importance within the human body [25]. Conjugates formed from the covalent bonding between FAs and active molecules represent a specific type of lipidic prodrugs in which, from a chemical perspective, the drugs containing hydroxyl groups are linked to the carboxylate group present within the FA’s molecule, leading to an ester-type linkage, one of the most fundamental chemical bonds found in various functional compounds, including pharmaceuticals [23]. Moreover, it has been previously revealed that drug esterification using FAs corrects its biopharmaceutical limitations by increasing drug stability, lipophilic nature, half-life, cell uptake, and passing across biological membranes [26]. In particular, unsaturated FAs (UFAs) became attractive for drug development, their attachment to active molecules leading to the production of efficient prodrugs [23]. Among all existing UFAs, oleic acid (OA) and linoleic acid (LA) are abundantly found in the human diet and are instilled with multiple functions in health, physiology, and nutrition [23, 25]. As regards the bioactivities of OA, its hepatoprotective properties and ability to alleviate liver disorders (e.g., fibrosis and cirrhosis) or chemically produced injury are well documented [27]. Additionally, OA remarks owing to its cardioprotective effects, reducing the risk for cardiovascular diseases [25], while also presenting a beneficial contribution to cutaneous health by modulating inflammation and enhancing the reparative response in skin wounds [28]. Comparatively, LA constitutes an essential FA, playing an important role in improving cardiac function, liver metabolic status and fat, and regulating cholesterol plasma levels [25]. Moreover, LA plays a significant contribution to the structural integrity of the skin through the formation of ceramides which are essential for the constitution of the cutaneous barrier [29]. Besides these benefits, UFAs also bear disadvantageous characteristics, such as instability and proneness to oxidative degradation [30].

Despite the tremendous research highlighting the therapeutical effects retained by SIL and its limited pharmacokinetics, the well-known health-promoting properties of UFAs and their oxidative instability, as well as the emerging trend in the fruition of UFAs in the obtainment of lipophilic drug complexes with improved physical, chemical, and pharmacological features, the evaluation of SIL-UFA derivatives in terms of biomedical uses remains scarce at present. Therefore, built upon previous reports suggesting the cardiac, hepatic, and cutaneous health benefits of SIL, OA, and LA, as well as the improved properties of the conjugates produced via the derivatization of active agents with UFAs, the current study proposed the synthesis via biocatalytic acylation, the comprehensive physicochemical characterization, and the in vitro–in ovo safety assessment of SIL oleate (SIL-O) and SIL linoleate (SIL-L) as prodrugs with potential applications in biomedicine with a specific direction towards heart, liver, and skin pathologies. The rationale behind the conjugation of SIL with OA and LA stands in the fact that this approach might result in lipophilic complexes with improved pharmacokinetic properties, stability, and therapeutic effect obtained through the enhancement of the flavolignan’s absorption and bioavailability following in vivo administration, the protection of the UFAs from potential oxidative damage, and the association of bioactive UFAs with a natural compound instilled with pharmacological activities.

## Materials and methods

### Reagents

The following reagents were provided by Sigma Aldrich, Merck KgaA (Darmstadt, Germany): SIL (HPLC, ≥ 98%), OA (GC, ≥ 99%), LA (GC, ≥ 98%), Novozyme 435 (Immobilized Candida antarctica lipase), methanol (AR, ≥ 99.5%), sodium dodecyl sulfate (GC, ≥ 98.5%), acetone (HPLC, ≥ 99.9%), chloroform (HPLC, GC, ≥ 99.9%), molecular sieves, insulin from bovine pancreas, hydrocortisone 21-hemisuccinate sodium salt (HPLC, ≥ 90%), and MTT (3-[4,5-dimethylthiazol2-yl]-2,5-diphenyltetrazolium) bromide reagent. Hoechst 33342 solution was bought from Invitrogen (Waltham, MA, USA). Dulbecco’s Modified Eagle Medium (DMEM; ATCC^®^ 30–2002™), trypsin-EDTA solution, phosphate saline buffer (PBS), dimethyl sulfoxide (DMSO), fetal calf serum (FCS), and penicillin/streptomycin were bought from ATCC (American Type Culture Collection, Lomianki, Poland). William’s Medium E was acquired from Gibco (Waltham, MA, USA).

### Regioselective synthesis of silibilin-O (SIL-O) and silibilin-L (SIL-L)

SIL derivatives were biosynthesized similarly to a method described in a recent publication [25]. SIL-O and SIL-L were produced by solubilizing SIL, OA, and LA in a reaction medium containing acetone (previously dried on 4-Å molecular sieves). The process was conducted for 10–12 h, at 50 ^∘^C, and under magnetic stirring (at a rate of 250 rpm). The esterification was initiated by the addition of the Novozyme 435 followed by a 120 h stirring using an orbital shaker, at 250 rpm and 50 ^∘^C. The used molar mass ratios of SIL:OA and SIL:LA were 1:3. The water content in the reaction was maintained under 0.3%. The formation of SIL-O and SIL-L was monitored using thin layer chromatography (TLC) and chloroform/ethyl acetate (60/40 v/v) as an eluent system, while their purification was performed by chromatographic column separation. Finally, the fractions that contained the SIL-O and SIL-L bioconjugates were introduced into a Loborota 4000 Efficient Rotary Evaporator (Heidolph Instruments, Schwabach, Germany) to remove the solvent, and their purification was achieved by recrystallization from acetone and methanol.

### Vibrational Fourier transform–Raman (FT–Raman) spectroscopy

The FT–Raman spectra of SIL, OA, LA, SIL-O, and SIL-L were obtained with an Equinox 55 Bruker spectrometer (Billerica, MA, USA) containing an integrated FRA 106S Raman module. The excitation was performed using a Nd:YAG laser operating at 1064 nm and with an output power of 350 mW, while the detection was ensured by a Ge detector operating at liquid nitrogen temperature. For data acquisition, a number of 350 scans at a spectral resolution of 2 cm^−1^ were collected.

### Density functional theory (DFT) calculations

Geometry optimizations and normal modes calculations have been performed in gas-phase, with the Gaussian 16, revision C.01 software [31] by using the DFT approximation at B3LYP/6-311+G(d,p) level of theory. The most stable conformers were identified for each of the investigated systems (SIL, SIL-O, SIL-L). For these calculations, we used the GFN2-xTB method [32] as implemented in the CREST software [33–35]. Subsequently, we re-optimized the first most stable five conformers for each system using the B3LYP/6-311+G(d,p) method. Finally, the most stable conformer of each system was used for normal mode calculations.

### ^1^H liquid-state nuclear magnetic resonance (NMR) spectroscopy

Measurements have been performed using a Bruker Avance 500 NMR spectrometer (Billerica, MA, USA) operating at an 11.75 T magnetic field. Samples were filled into 5 mm NMR tubes and the temperature was maintained at 300 K. Deuterated dimethyl sulfoxide (DMSO-d6) was used as a solvent in all the investigated samples. A number of 32 scans were accumulated, and the relaxation delay was set to 3 s.

### Computational predictions of drug-likeness and toxic potential

To predict the drug-like properties and potential toxic effects of SIL, SIL-O, and SIL-L, the open-source program OSIRIS Property Explorer was employed which estimates a molecule’s mutagenic, tumorigenic, irritant, and reproductive toxicity, as well as the molecular weight (MW), cLogP, solubility, drug-likeness, and drug score based on its chemical structure [36].

### In vitro 2D models

The study was performed using three cell lines – H9c2(2-1) cardiomyoblasts (CRL-1446™), HepaRG hepatocytes (HPRGC10), and HaCaT keratinocytes (300493) – delivered as frozen vials by ATCC (Lomianki, Poland), ThermoFisher Scientific (Waltham, MA, USA), and CLS Cell Lines Service GmbH (Eppelheim, Germany), respectively. H9c2(2-1) and HaCaT cells were grown in DMEM, while HepaRG cells were cultured in William’s E Medium containing insulin from bovine pancreas (at a final concentration of 4 µg/mL) and hydrocortisone 21-hemisuccinate sodium salt (at a final concentration of 50 µM). Both media were supplemented with 10% FCS and 1% antibiotics (penicillin 100 U/mL and streptomycin 100 µg/mL). The cells were cultured at 37 ^∘^C and 5% CO_2_, in a humidified incubator for cell culture, presenting normal proliferation during the experiments.

### Cell viability assay

The impact of all tested compounds (1, 10, 25, 50, and 100 µM) on the viability of H9c2(2-1), HepaRG, and HaCaT cells was assessed using the MTT method which was performed according to a previous publication [37]. Briefly, the cells cultured in 96-well plates were exposed to SIL, SIL-O, and SIL-L for 24 h. At the end of the treatment, the culture medium containing SIL, SIL-O, and SIL-L was removed and replaced with 100 µL/well of the specific culture media for the used cell lines (DMEM for H9c2(2-1) cells and HaCaT; William’s E Medium for HepaRG). Then, 10 µL of MTT reagent was added in every well, followed by 3 h of incubation of the plates at 37 ^∘^C. The absorbance measurements were performed at 570 and 630 nm on Cytation 5 Microplate Reader (BioTek Instruments Inc., Winooski, VT, USA) following the addition of the MTT solubilization solution (a volume of 100 µL/well), and the plates’ incubation at room temperature for 30 min, protected from light.

### Cell morphology and confluence evaluation

To verify whether SIL and its derivatives (100 µM) affect healthy cells’ morphology, a bright-field microscopical evaluation was performed after a 24-h treatment. The images were analyzed using Cytation 1 Imaging Reader (BioTek Instruments Inc., Winooski, VT, USA). Cell confluence (%) was automatically measured using the Image Analysis tool provided by Gen5™ Microplate Data Collection and Analysis Software (BioTek Instruments Inc., Winooski, VT, USA), and according to the manufacturer’s recommendations.

### Hoechst 33342 nuclear staining

The influence of SIL, SIL-O, and SIL-L (100 µM) on nuclear morphology was evaluated by imaging the cells’ nuclei stained with Hoechst 33342. Shortly, at the end of the treatment, the H9c2(2-1), HepaRG, and HaCaT cells cultured in 12-well plates were treated with staining solution (500 µL/well, dilution of 1:2000 in PBS) for 10 min at room temperature, protected from light and washed with PBS before imaging which was performed on Cytation 1 Imaging Reader (BioTek Instruments Inc., Winooski, VT, USA). Image processing was conducted using the Gen5™ Microplate Data Collection and Analysis Software (BioTek Instruments Inc., Winooski, VT, USA). Apoptotic index (AI) was calculated by applying the formula previously reported [38]:


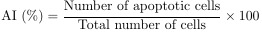


### Cell migration assay

To investigate the effect of SIL, SIL-O, and SIL-L treatments on the migration of H9c2(2-1), HepaRG, and HaCaT cells, the scratch assay was performed as follows: (A) the cells (10^5^ cells/mL/well) were seeded in Corning Costar 24-well plates and left to attach and reach a proper confluence, (B) a scratch was automatically made in every well using the AutoScratch™ Wound Making Tool (BioTek Instruments Inc., Winooski, VT, USA), (C) the cells were exposed to SIL and its FAs derivatives 1, 10, and 25 µM for 24 h, and (D) the wound area was imaged at two time points (0 and 24 h) using Cytation 1 Imaging Reader (BioTek^®^ Instruments Inc., Winooski, VT, USA). The Gen5 ™ Microplate Data Collection and Analysis Software (BioTek^®^ Instruments Inc., Winooski, VT, USA) was used to measure the wound widths in each image. The migration rates (%) for the applied treatments were determined using the formula presented below and normalized to control.

Migration rate (%) ═ 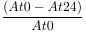 ×100, where:

At_0_ ═ wound width at 0 h,

At_24_ ═ wound width at 24 h.

### In ovo irritation test

The potential irritant effect of SIL-O and SIL-L (100 µM) was assessed in ovo, by applying the Hen’s Egg Test – Chorioallantoic Membrane (HET-CAM) test. The applied procedure was also described by Breban-Schwarzkopf et al. [39]. Briefly, chicken-fertilized eggs were cleaned and disinfected with ethanol 70% and placed in an incubator at 37 ^∘^C and 60% humidity. On the fourth day of incubation, a cut was performed on the eggs’ tips to allow the extraction of 6–7 mL albumen, while on the fifth day, a window was cut on the upper side of each egg and covered with adhesive tape. The experiment was performed on the tenth day of incubation, by applying the samples directly on the chorioallantoic membrane (CAM) and analyzing the occurrence of vascular impairments, such as hemorrhage, lysis, and coagulation for 5 min. Representative pictures were taken before the start of the experiment (T0) and at its end (T5) using the Discovery 8 SteREO Microscope equipped with an Axio CAM 105 color camera and processed in ZEN core version 3.8 software (Zeiss, Göttingen, Germany). The irritant potential was determined by calculating the irritation score (IS) for each sample and interpreted as presented in a previous publication [40].

### Ethical statement

The present study did not require an ethical approval.

### Statistical analysis

The data from this paper are presented as mean ± standard deviation (SD). The differences between data were compared in GraphPad Prism 9.2.0 version for Windows (GraphPad Software, San Diego, CA, USA, www.graphpad.com), using the one-way ANOVA analysis and Dunett’s multiple comparisons post-test. Statistical significance was marked with (*P < 0.05, **P < 0.01, ***P < 0.001, ****P < 0.0001 vs control).

**Figure 1.**
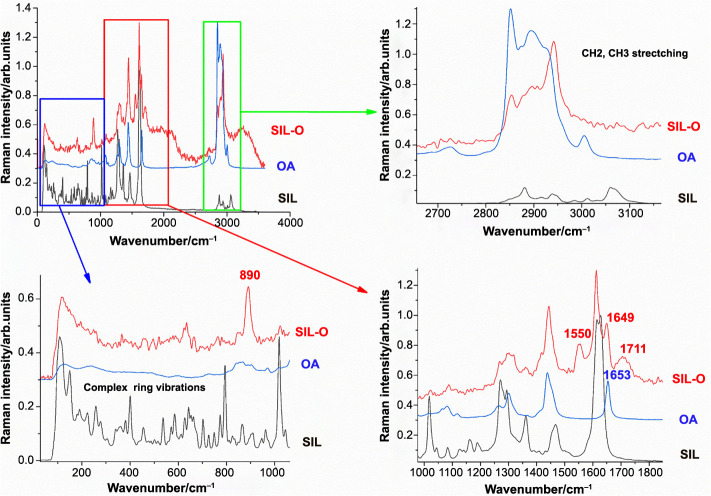
**Comparative FT–Raman spectra of silibinin, oleic acid, and silibinin oleate.** Excitation: 1064 nm. SIL: Silibinin; OA: Oleic acid; SIL-O: Silibinin oleate.

**Figure 2.**
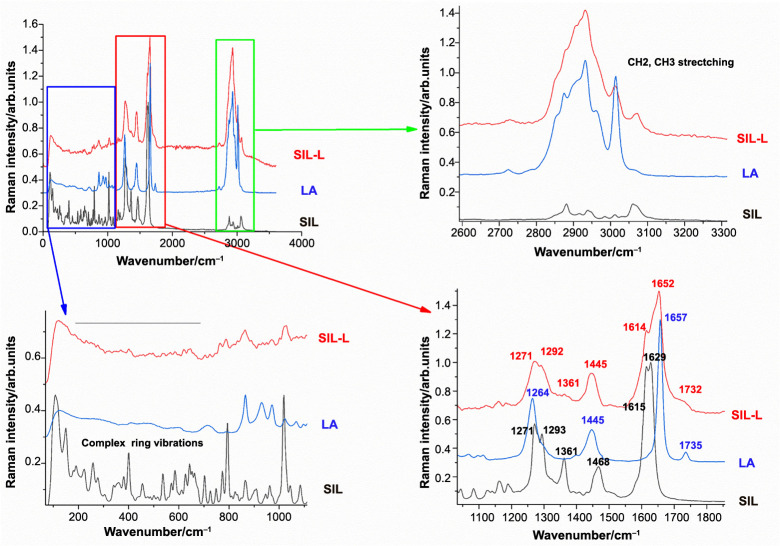
**Comparative FT–Raman spectra of silibinin, linoleic acid, and silibinin linoleate.** Excitation: 1064 nm. SIL: Silibinin; LA: Linoleic acid; SIL-L: Silibilin linoleate.

**Figure 3.**
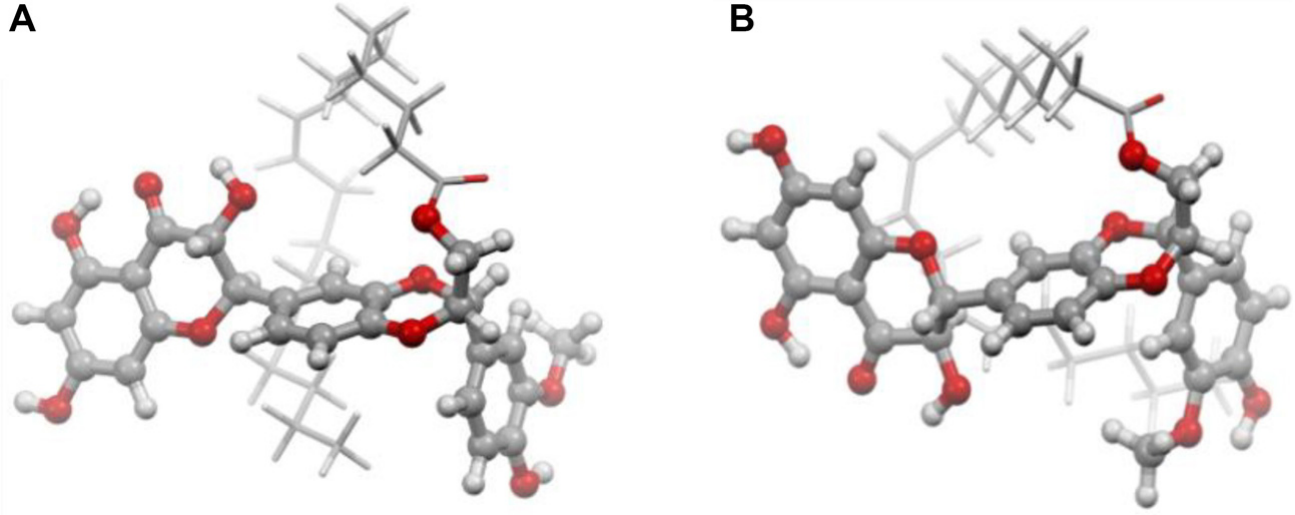
**3-D molecular structure of the most stable (A) silibinin oleate and (B) silibinin linoleate**.

## Results

### Vibrational spectroscopy of SIL, SIL-O and SIL-L

The specific FT–Raman spectra for parent compounds (SIL, OA, and LA) and the resulting derivatives (SIL-O, SIL-L) are presented in Figures 1 and 2. The main Raman bands of OA can be found in the 1200–1700 cm^−1^ region, at 1265 cm^−1^, (═C–H) deformation of cis(R–HC=CH–R), at 1300 cm^−1^, which can be assigned to (C–H) bending twist of the CH_2_ group, at 1440 cm^−1^ attributed to (C–H) scissoring of CH_2_ and at 1655 cm^−1^, assigned to (C═C) stretching of cis(RHC=CHR). In the case of the LA small shifts are visible in the spectra compared to OA, the band corresponding to the (C–H) scissoring vibration appears at 1450 cm^−1^ while the (C═C) stretching band is present at 1657 cm^−1^. Moreover, LA presents an additional band, not visible in OA at 1735 cm^−1^, attributed to the (C═O) stretching of RC=OOR group [41, 42]. In the high wavenumber region, 2800–3200 cm^−1^, several bands are visible in the spectra of both acids, attributed to the C–H stretching modes, with differences in their spectra both in terms of band intensity and position. The main bands of OA are at 2727, 2848, 2893, and 3003 cm^−1^, while the LA bands are located at 2848, 2875, 2893, 2931, 2961, and 3014 cm^−1^. The spectra of oleate/linoleate derivatives are dominated by the characteristic bands of the parent compounds, with several small changes, mainly characteristic of the vibrational modes change on passing from SIL to its corresponding derivatives. For SIL-O, the same behavior can be observed in the high wavenumber (∼3000 cm^−1^) region, however, in the low wavenumber region 0–2000 cm^−1^ region three bands were recorded, which cannot be found in the spectra of SIL or OA at 890, 1551, and 1711 cm^−1^; the 1711 cm^−1^ peak can be assigned to the stretching of the C=O bond of the ester functional group. The other peaks visible in the SIL-O can be attributed to either SIL or OA, with the position of the peaks slightly shifted. To be noted, the characteristic C=C stretching band is also shifted from 1655 cm^−1^ in OA to 1648 cm^−1^ in SIL-O. In the high wavenumber 2800–3200 cm^−1^ region the visible SIL-O bands, corresponding to the C-H stretching modes, are generated by the OA and SIL with small shifts and changes in relative intensity. Moreover, likely as a result of esterification, the SIL-O spectrum is missing some of the intense bands present in the parent compounds: 3005 cm^−1^ of OA or 3059 cm^−1^ of SIL. Opposed to SIL-O, in the SIL-L, all the bands present in the 1000–2000 cm^−1^ spectral region have corresponding bands either in SIL or in the LA, albeit with slight shifts. The C═C band appears at 1652 cm^−1^ in SIL-L as opposed to 1657 cm^−1^ in LA, while the band corresponding to the C═O stretch found at 1735 cm^−1^ in LA appears as a weak shoulder at 1732 cm^−1^.

### DFT analysis of SIL, SIL-O, and SIL-L

Conformational analysis of the individual molecules (SIL, and the linoleate and oleate esters SIL-O and SIL-L) resulted in a set of five conformers. The molecular structures of the most stable conformers are given in Figure 3. The comparison of the position of the experimental and theoretical DFT Raman bands is shown in Table 1. SIL-L and SIL-O complexes can be distinguished by the bands in the high Raman shifts in the 2800–3100 cm^−1^ spectral range, these changes seem to confirm the esterification of SIL. In the fingerprint spectral range, the **ν**(C ═ C) bands appear at different positions (1652 cm^−1^ for SIL-L and 1649 cm^−1^ for SIL-O). The band corresponding to the **ν**(CH)r4 vibrational mode, which appears at 3100 cm^−1^ SIL-O and SIL-L in the calculations, is expected to reduce in intensity due to the connection of the linoleate or oleate side chain. Accordingly, the corresponding band is not visible in the experimental spectrum of SIL-L and is very weak in the experimental spectrum of SIL-O. Moreover, the DFT calculations predict that the **ν**(C-CH_2_OH)r4 vibration mode at 1361 cm^−1^ is likely to disappear as a direct consequence of the esterification, in the experimental spectra, this band is present but with very weak intensity. Overall, the comparison of the vibrational theoretical and experimental Raman data showed a good agreement, and thus, the changes observed in the SIL-O and SIL-L Raman spectra compared to that of SIL, strongly support the evidence of the derivative structures’ formation. Detailed information and theoretical DFT Raman spectra comparison are given in Figures S1 and S2. The agreement between theory and experiment supports the proof of SIL derivatives formation.

**Figure 4.**
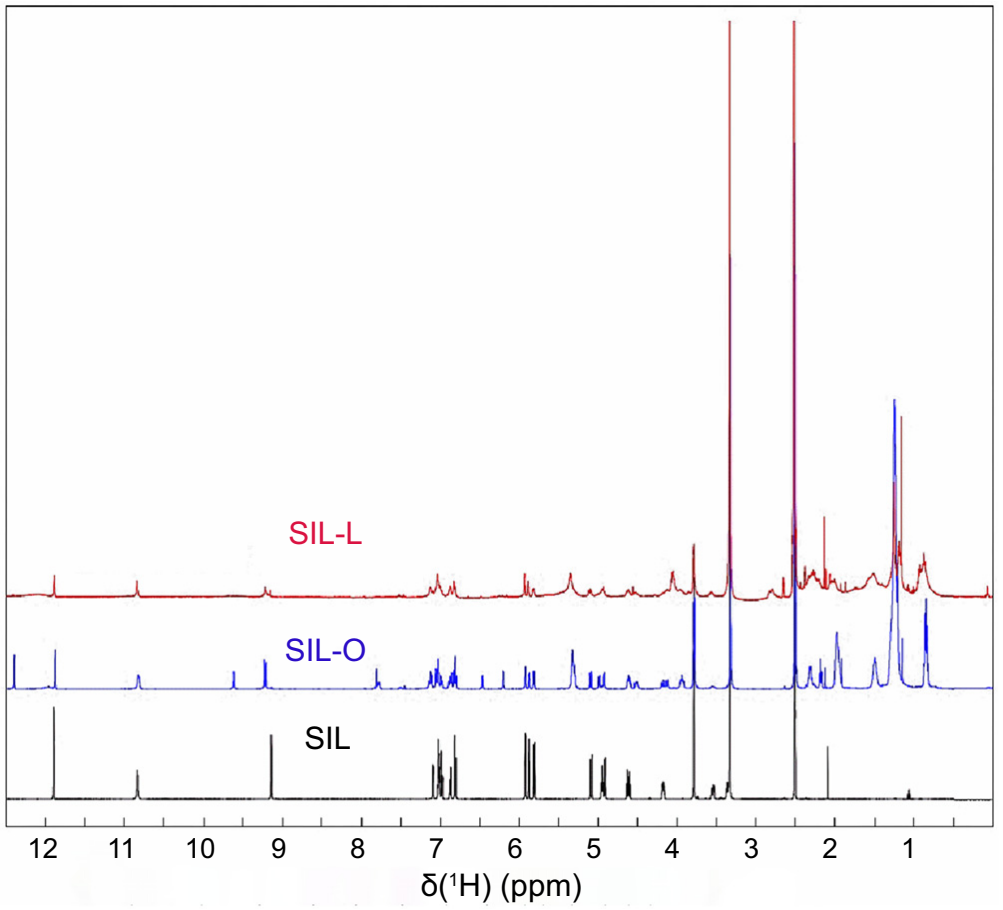
**^1^H NMR spectra of silibinin, silibinin oleate, and silibinin linoleate.** SIL: Silibinin; SIL-O: Silibinin oleate; SIL-L: Silibilin linoleate.

### ^1^H liquid state NMR spectroscopy of SIL, SIL-O, and SIL-L

Figure 4 shows a comparison between the 1H liquid state NMR spectra of SIL, SIL-O, and SIL-L. Their chemical structures and peak assignment are presented in Table S1 accordingly, taking into account previous data from the literature on SIL, OA, and LA [28–31]. To validate the esterification process, interactions between the OH- groups of SIL and protons of the carboxylic acid of OA and LA, respectively, were investigated. It was found that only the intensity of protons from the carboxylic acid of OA and LA is reduced six times in both ^1^H NMR spectra of SIL-O and SIL-L, showing up as a doublet peak centered at 9.19 ppm, respectively. Moreover, the peak corresponding to the proton of the carboxylic group of the OA and LA has a low intensity in the former case and is missing from the ^1^H NMR spectrum in the latter case. Additionally, a signal corresponding to water was found at 4.51 ppm in the ^1^H NMR spectra of SIL-O and SIL-L. Moreover, new peaks were identified in these spectra, as indicated in Table S1. These results are in concordance with the experimental and theoretical Raman spectra and prove that the esterification process occurs between the hydroxyl group attached to the a4” carbon (equivalent to the C-23 position from the literature [32]) of SIL (the molecular structure is presented in Table S1) and the proton of the COOH group of the respective acids.

**Table 1:** Experimental and DFT calculated Raman bands (wavenumber/cm^−1^) of silibilin, silibinin oleate and silibinin linoleate

**Mode**	**SIL**			**SIL-O**			**SIL-L**		
	**Exp.**	**Calc.**	**Assign.**	**Exp.**	**Calc.**	**Assign.**	**Exp.**	**Calc.**	**Assign.**
Q1	3071	3194	**ν**(CH)_r5_	3073	3194	**ν**(CH)_r5_	3072	3193	**ν**(CH)_r5_
Q2	3061	3182	**ν**(CH)_r1_	3060	3182	**ν**(CH)_r1_	3064	3182	**ν**(CH)_r1_
	3012	3139	**ν**_as_(CH_3_)	3018	3141	**ν**_as_(CH_3_)	3012	3140	**ν**_as_(CH_3_)
Q3	2985	3095	**ν**_as_(CH_2_)+**ν**(CH)_r4_	2983 vw	3100	**ν**(CH)_r4_	–	3099	**ν**(CH)_r4_
Q4	–	–	–	2970	3070	**ν**(CH)_r4_+**ν**(CH_2_)	2970	3072	**ν**(CH)_r4_+**ν**(CH_2_)
Q5	2946	3082	**ν**_as_(CH_2_)+**ν**(CH)_r4_oop_	2941	3070	**ν**_as_(CH_2_)+**ν**(CH)_r4_	–	3060	**ν**_as_(CH_2_)−**ν**(CH)_r4_
Q6	2939	3074	**ν**_as_(CH_2_)+**ν**(CH)_r4_ip_	2930	3059	**ν**_as_(CH_2_)−**ν**(CH)_r4_	2931	3072	**ν**_as_(CH_2_)+**ν**(CH)_r4_
Q7	2916	3023	**ν**(CH)_r2_	–	–	–	2916	3027	**ν**(CH)_r2_
Q8	–	–	–	2906	3025	**ν**_as_(CH_2_)_ole_	2906	3026	**ν**_as_(CH_2_)_lin_
Q9	–	–	–	2897	3023	**ν**_s_(CH_2_)_ole_	–	3003	**ν**_s_(CH_2_)_lin_
Q10	2881	3016	**ν**_s_(CH_3_)	2877	3015+3017	**ν**_s_(CH_3_)_ole_+ **ν**_s_(CH_3_)_slbn_	2881	3018+3016	**ν**_s_(CH_3_)_slbn_+**ν**_s_(CH_3_)_lin_
Q11	2862	3012	**ν**_s_(CH_2_)	–	–	–	2859	3014	**ν**_s_(CH_2_)_slbn_+**ν**_as_(CH_2_)_lin_
Q12	2850	2954	**ν**(CH)_r2_	2845	2955	**ν**(CH)_r2_	2850	2965	**ν**(CH)_r2_
Q13	–	–	–	1717	1786	**ν**(C═O)	1732	1786	**ν**(C═O)
	–	–	–	1649	1709	**ν**(C═C)_ole_	1652	1715+1709	**ν**(C═C)_lin_ip_+ **ν**(C═C)_lin_oop_
Q14	1628	1675	**ν**(C═C)_r1_+**ν**(C═O)_r2_	1621	1675	**ν**(C═C)_r1_+**ν**(C═O)_r2_	1639	1674	**ν**(C═C)_r1_+ **ν**(C═O)_r2_
Q15	1614	1651	**ν**(CC)_r3_	1612	1652	**ν**(CC)_r3_	1614	1650	**ν**(CC)_r3_
Q16	1468	1497	**ν**(CO)_r1r2_+δ(CCC)_r1r2_	1444	1498	**ν**(CO)_r1r2_+δ(CCC)_r1r2_	1444	1497	**ν**(CO)_r1r2_+ δ(CCC)_r1r2_
Q17	1361	1425	**ν**(C−CH_2_OH)_r4_	–	–	–	–	–	–
Q18	1294	1283	def.(r4,r5)	1302	1326	def.(r4,r5)	1290	1288	def.(r4,r5)
Q19	1271	1276	δ(CCC)_r3_	1269	1277	δ(CCC)_r3_	1271	1280	δ(CCC)_r3_
Q20	1018	1035	δ(CCC)_r1_+δ(OCC)_r2_	1022	1038	δ(CCC)_r1_+δ(OCC)_r2_	1028	1030	δ(CCC)_r1_+ δ(OCC)_r2_
Q21	792	794	δ(OCC)_r2_+δ(CCC)_r3_	–	–	–	789	804	δ(OCC)_r2_+ δ(CCC)_r3_
Q22	401	394	def.(r1,r2)	–	–	–	–	–	–

SIL: Silibinin; SIL-O: Silibinin oleate; SIL-L: Silibinin linoleate; Exp: Experimental; Calc.: Calculated; Assign: Assignments.

**Table 2:** Computational prediction of silibinin, silibinin oleate, and silibinin linoleate properties obtained using OSIRIS Property Explorer

**Compound**	**MW**	**cLogP**	**Solubility**	**Drug-likeness**	**Drug score**	**Mutagenic, tumorigenic, irritant and reproductive toxicity**
SIL	482	2.13	−3.41	1.64	0.64	No risk
SIL-O	746	9.63	−7.91	−26.96	0.07	
SIL-L	744	9.38	−7.68	−23.55	0.07	

SIL: Silibinin; SIL-O: Silibinin oleate; SIL-L: Silibinin linoleate; MW: Molecular weight; cLogP: Logarithm of partition coefficient between n-octanol and water.

### Drug-likeness and toxic potential of SIL, SIL-O, and SIL-L

The MW, logarithm of partition coefficient between n-octanol and water (cLogP), solubility, drug-likeness, drug score, and potential toxicity of SIL, SIL-O, and SIL-L were computationally determined using the OSIRIS Property Explorer program, and presented in Table 2. According to the results, SIL-O and SIL-L present higher MW and cLogP values, but also a lower solubility compared to their parent compound SIL. Moreover, both derivatives presented a negative drug-likeness and a lower drug score than SIL. When comparing the parameters predicted for SIL-O and SIL-L, it was observed that both present the same positive drug score value, however, SIL-L has a higher drug-likeness compared to SIL-O. The investigated compounds presented no predicted risk for mutagenic, tumorigenic, irritant, or reproductive toxicity.

### Impact of SIL, SIL-O, and SIL-L on cellular viability

**Figure 5.**
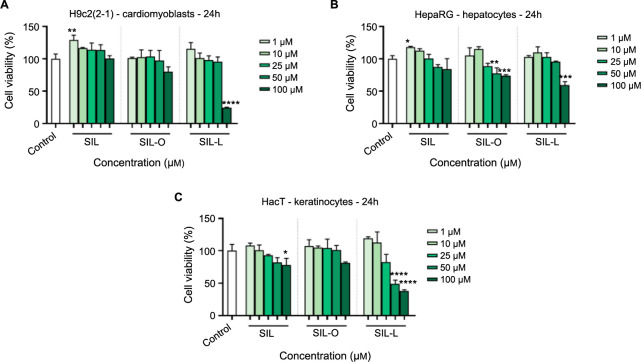
**In vitro assessment of the impact exerted by silibinin, silibinin oleate, and silibinin linoleate at five concentrations (1, 10, 25, 50, and 100 µM) on the viability of healthy (A) cardiomyoblasts—H9c2(2-1), (B) hepatocytes—HepaRG, and (C) keratinocytes—HaCaT following a 24 h treatment.** The data are presented as viability percentages (%) normalized to control (untreated cells) and are expressed as mean values ± SD of three independent experiments performed in triplicate. The statistical differences between the control and the treated groups were verified by applying the one-way ANOVA analysis followed by Dunnett’s multiple comparisons post-test (*P < 0.05; **P < 0.01; ***P < 0.001; ****P < 0.0001 versus control). SIL: Silibinin; SIL-O: Silibinin oleate; SIL-L: Silibilin linoleate; SD: Standard deviation.

To verify the potential cytotoxicity of SIL-O and SIL-L on cardiac, hepatic, and skin cells (H9c2(2-1), HepaRG, HaCaT), an MTT assay was performed. Comparatively, the parent compound SIL was also tested. As presented in Figure 5, a gradual and dose-dependent decrease in the viability of all cell lines was noticed upon their 24-h treatment with SIL, SIL-O, and SIL-L. However, the effect was highly dependent on the tested compound and cell type. In H9c2(2-1) cells, SIL showed a stimulatory effect on cell viability, all percentages being over 100% compared to control at the tested concentrations, although statistical significance was reached only at 1 µM when the viability presented a value of 128.4%. SIL-O and SIL-L also stimulated the viability of H9c2(2-1) cells but only at low concentrations (up to 25 µM – SIL-O, and up to 10 µM – SIL-L), while gradually reducing it at higher ones. The lowest viability value (24.72%) was obtained following the exposure of cardiomyoblasts to SIL-L 100 µM. Similar effects were observed in the case of HepaRG cells whose viability was stimulated by the treatment with SIL (1, 10, and 25 µM), SIL-O (1 and 10 µM), and SIL-L (1, 10, and 25 µM). Higher concentrations led to a reduction in the percentage of HepaRG viable cells up to around 80% (SIL), 70% (SIL-O), and 60% (SIL-L), respectively. In HaCaT keratinocytes, stimulation of cell viability was noticed in the case of all tested compounds, but only at low concentrations (up to 10 µM – SIL and SIL-L; up to 50 µM – SIL-O). The highest cytotoxicity was induced by SIL-L 50 and 100 µM, the viability percentages being 48.95% and 38.22%, respectively.

### Impact of SIL, SIL-O, and SIL-L on cellular morphology and confluence

**Figure 6.**
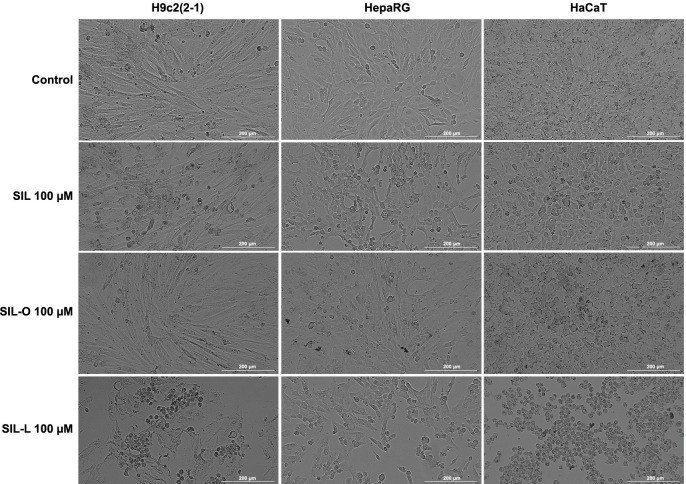
**Bright-field images illustrating the morphology of cardiomyoblasts** – **H9c2(2-1), hepatocytes** – **HepaRG, and keratinocytes** – **HaCaT following a 24-h treatment with silibinin, silibinin oleate, and silibinin linoleate at 100 µM.** The scale bars indicate 200 µm. SIL: Silibinin; SIL-O: Silibinin oleate; SIL-L: Silibilin linoleate.

**Figure 7.**
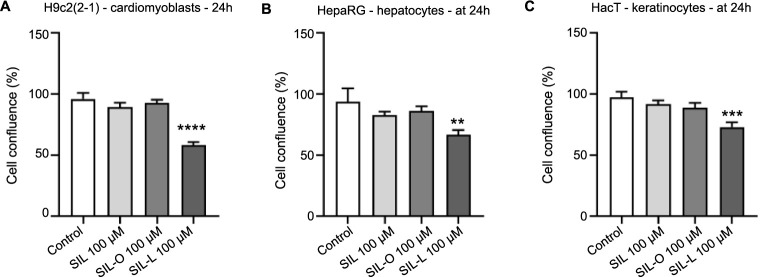
**Cell confluence determination in (A) cardiomyoblasts** – **H9c2(2-1), (B) hepatocytes** – **HepaRG, and (C) keratinocytes** – **HaCaT following a 24 h treatment with silibinin, silibinin oleate, and silibinin linoleate at 100 µM using the Cell Analysis tool provided by the Gen5 Microplate Data Collection and Analysis Software (*N* ═ 3).** The statistical differences between the control and the treated groups were verified by applying the one-way ANOVA analysis followed by Dunnett’s multiple comparisons post-test (**P < 0.01; ***P < 0.001; ****P < 0.0001). SIL: Silibinin; SIL-O: Silibinin oleate; SIL-L: Silibilin linoleate.

Further, the impact of SIL, SIL-O, and SIL-L 100 µM on cells’ morphological features was assessed (Figure 6). Apart from a slight reduction in the confluence of HepaRG cells, SIL and SIL-O caused no alterations in the morphology of healthy cell lines compared to the control. The most significant morphology changes (e.g., rounding, shrinkage, detachment from the plate, and debris) which are indicative of cytotoxicity were observed only after the H9c2(2-1), HepaRG, and HaCaT cells’ 24-h treatment with SIL-L. As presented in Figure 7, the confluence of H9c2(2-1), HepaRG, and HaCaT cells treated with SIL and SIL-O were similar to control, while significant reductions were determined in the case of the cells treated with SIL-L (to 58.23% – H9c2(2-1), 66.7% – HepaRG, and 73.06% – HaCaT), these data supporting the obtained viability and cell morphology results which indicate that SIL-L (100 µM) exerts the highest cytotoxicity among the tested compounds.

### Impact of SIL, SIL-O, and SIL-L on nuclear morphology

**Figure 8.**
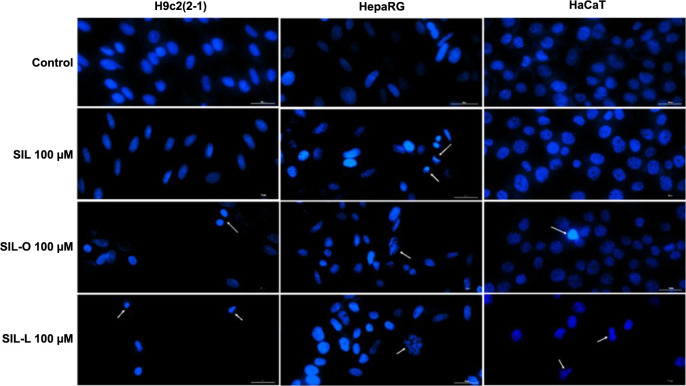
**Nuclear staining of cardiomyoblasts** – **H9c2(2-1), hepatocytes** – **HepaRG, and keratinocytes** – **HaCaT following a 24 h treatment with silibinin, silibinin oleate, and silibinin linoleate at 100 µM.** White arrows indicate nuclei presenting an apoptotic-specific aspect. The scale bars represent 30 µm. SIL: Silibinin; SIL-O: Silibinin oleate; SIL-L: Silibilin linoleate.

**Figure 9.**
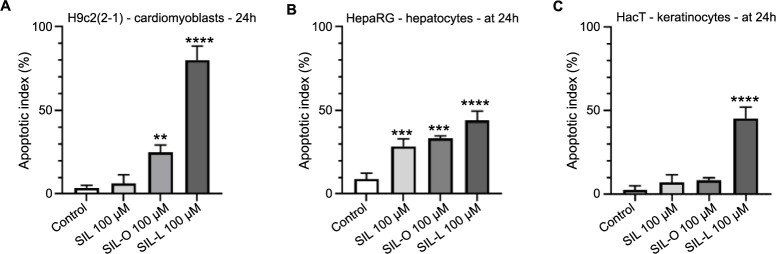
**Apoptotic index (%) calculation in Hoechst 33342-stained (A) H9c2(2-1) cardiomyoblasts, (B) HepaRG hepatocytes, and (C) HaCaT keratinocytes after a 24 h treatment with silibinin, silibinin oleate, and silibinin linoleate 100 µg/mL.** The data are presented as apoptotic index (%) expressed as mean values ± SD of three independent experiments performed in triplicate. The statistical differences between the control and the treated groups were verified by applying the one-way ANOVA analysis followed by Dunnett’s multiple comparisons post-test (**P < 0.01; ***P < 0.001; ****P < 0.0001 vs control). SIL: Silibinin; SIL-O: Silibinin oleate; SIL-L: Silibilin linoleate; SD: Standard deviation.

To further investigate the possible cell death mechanism underlying the cytotoxicity of SIL, SIL-O, and SIL-L 100 µM in H9c2(2-1), HepaRG, and HaCaT cells, a Hoechst 33342 staining highlighting the nuclear morphological features was performed. Figure 8 presents the aspect of control nuclei which possess a well-defined shape (round or oval) and uniform chromatin distribution, as well as the apoptotic-like changes (white arrows) induced by SIL, SIL-O, and SIL-L at the highest concentration (100 µM). Compared to control, both derivatives (SIL-O and SIL-L) induced nuclear deformation, fragmentation, and chromatin condensation in all cell lines. SIL, however, induced nuclear changes such as condensation and deformation only in HepaRG cells. Compared to Control, a significant increase in AI (Figure 9A) was obtained in H9c2(2-1) cells treated with SIL-O and SIL-L (to 25% and 80%, respectively), while in HepaRG cells (Figure 9B), all tested compounds elevated the percentage of apoptotic-like nuclei, the highest increase being induced by SIL-L (to 44%). In HaCaT keratinocytes (Figure 9C), only SIL-L treatment increased the AI (to 45%), while SIL and SIL-O caused no significant changes compared to Control.

### Impact of SIL, SIL-O, and SIL-L on cell migration

**Figure 10.**
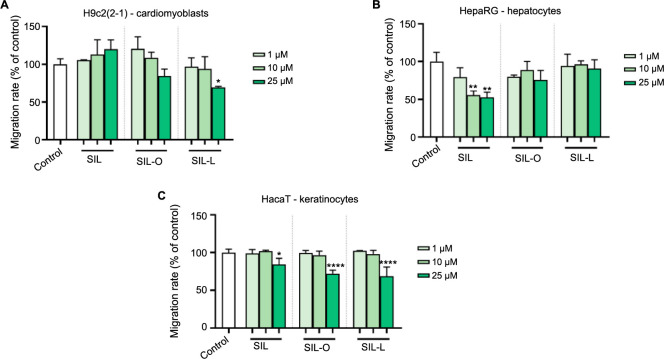
**In vitro assessment of the impact of silibinin, silibinin oleate, and silibinin linoleate (1, 10, and 25 µM) on the migration rate in (A) H9c2(2-1), (B) HepaRG, and (C) HaCaT cells following a 24 h treatment.** The data were normalized to control and are expressed as mean values ± SD of three independent experiments performed in triplicate. The statistical differences between the control and the treated groups were quantified by applying the one-way ANOVA analysis followed by Dunett’s multiple comparisons post-test (*P < 0.05; **P < 0.01; ****P < 0.0001). SIL: Silibinin; SIL-O: Silibinin oleate; SIL-L: Silibilin linoleate; SD: Standard deviation

To investigate whether the treatment with SIL, SIL-O, and SIL-L (1, 10, and 25 µM) impairs the migration of H9c2(2-1), HepaRG, and HaCaT cells, a scratch assay was performed at 24 h post-stimulation. The migration rates obtained are graphically shown in Figure 10, while the representative images taken at 0 and 24 h are shown in Figures S3–S5. The results indicated that the effects on cell migration were highly dependent on the tested compound, cell type, and applied concentration. In the case of H9c2(2-1) cells, it was found that SIL promoted the cells’ migration in a concentration-dependent manner, from 105.34% (at 1 µM) to 119.8% (at 25 µM). SIL-O also stimulated the migratory capacity of H9c2(2-1) cells, but only at concentrations of 1 and 10 µM (to 120.36% and 108.7%, respectively), while reducing it at 25 µM (to 84.43%). SIL-L induced no impairment in the migration rate of H9c2(2-1) cells at 1 and 10 µM (the migration rates were over 90%), but significantly reduced their motility at 25 µM (to 69.63%). In HepaRG cells, the highest inhibition in the migration rate was obtained following their treatment with SIL 10 and 25 µM (to values around 50%). SIL-O reduced the HepaRG cells’ migration at 25 µM (to 75.84%), while SIL-L caused no significant changes in their migratory ability. At the lowest tested concentrations (1 and 10 µM), neither SIL, nor SIL-O or SIL-L affected the migration of HaCaT cells, however, significant inhibition compared to control was obtained at the concentration of 25 µM for all tested compounds.

### In ovo irritant potential of SIL, SIL-O, and SIL-L

**Figure 11.**
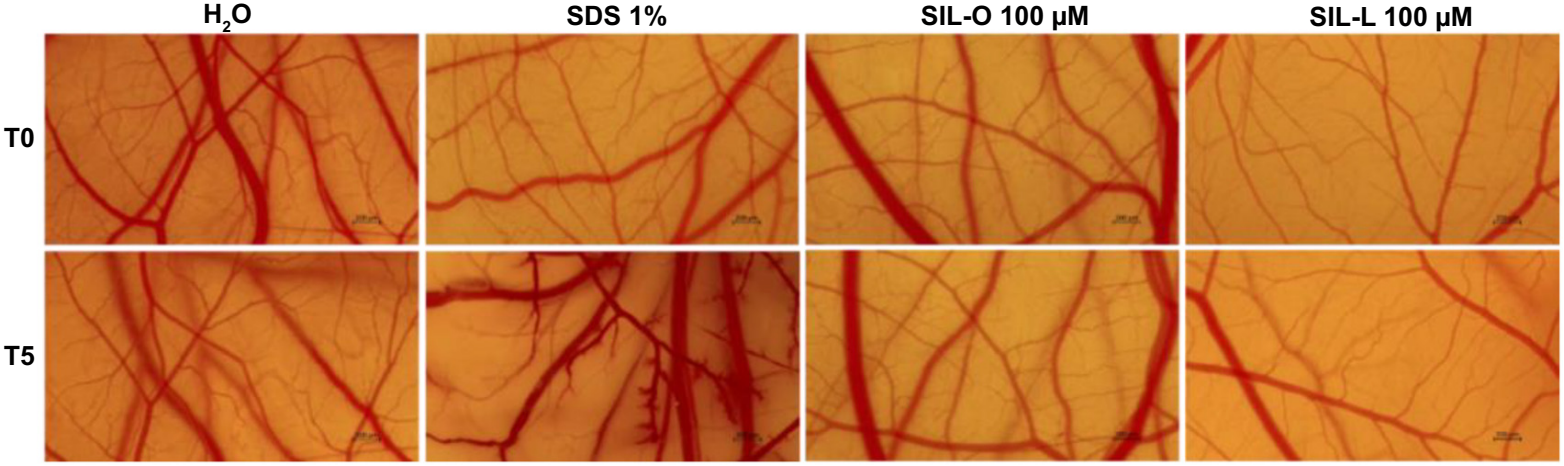
**Representative images of the chorioallantoic membrane captured before (T0) and 5 minutes (T5) after the treatment with H_2_O (water; negative control), SDS 1% (sodium dodecyl sulfate; positive control), silibinin oleate 100 µM and silibinin linoleate 100 µM.** The scale bars indicate 200 µm. CAM: Chorioallantoic membrane; SIL-O: Silibinin oleate; SIL-L: Silibilin linoleate.

The potential irritant effect of SIL-O and SIL-L at the highest tested concentration (100 µM) was explored in ovo, by applying the HET-CAM assay. The aspect of the CAM treated with H_2_O, SDS 1%, SIL-O, and SIL-L is presented in Figure 11. SIL-O caused no significant impairment on the vascular structure apart from slight signs of lysis. Comparatively, SIL-L induced microhemorrhage, but only at the end of the treatment. Based on the calculated IS values (Table 3), SIL-O induced no irritant effects on the CAM, while SIL-L was classified as a slight irritant. The most rapid and the most significant vascular toxicity was caused by SDS 1% used as a standard irritant.

**Table 3:** Irritation score values for water (H_2_O), sodium dodecyl sulfate 1% (SDS 1%), silibinin oleate, and silibinin linoleate 100 µM

**Sample**	**IS value**	**Observation**
H_2_O	0.07	Non-irritant
SDS 1%	19.74	Strong irritant
SIL-O 100 µM	0.61	Non-irritant
SIL-L 100 µM	1.99	Slight irritant

IS: Irritation score; SIL-O: Silibinin oleate; SIL-L: Silibilin linoleate.

## Discussion

SIL represents the most bioactive compound found in the silymarin complex of *S. marianum* L., exerting numerous health-improving properties, but limited stability and a poor pharmacokinetic profile, that hinders its therapeutic applications [7, 10, 18–20, 43]. A positive impact on the bioavailability and/or pharmacological effects of SIL was previously obtained through its conjugation with lipophilic moieties (e.g., phosphatidylcholine, and FAs) [24], however, the pharmaco-toxicological evaluation of lipidic SIL conjugates for potential biomedical applications remains scarcely covered at present. Therefore, the current study first proposed the derivatization of SIL using UFAs to produce oleate and linoleate conjugates (SIL-O and SIL-L) and their comprehensive physicochemical characterization, considering that the development of lipidic drug conjugates emerged as an effective approach that protects fragile drug moieties from extensive degradation and metabolization in vivo while increasing their lipophilicity which facilitates drug crossing through bilayer membranes via passive diffusion and increases drug bioavailability [23]. Additionally, taking into account the skin-, cardio-, and hepato-protective activities of SIL, OA, and LA [17, 28–30, 44], another main interest of this research was the in vitro–in ovo safety screening of SIL-O and SIL-L as potential ester-type prodrugs used for preventive or curative purposes in heart, liver, or skin disorders.

The study debuted with the biosynthesis of SIL-O and SIL-L conjugates through enzymatic acylation – an easy, inexpensive, and regioselective method [25]. The process was mediated using immobilized Candida antarctica lipase (Novozym^®^ 435) as a biocatalyst, considering that one of the primary applications of lipases is the synthesis of esters [45], it was already successfully used for the separation of SIL stereoisomers (A and B) [43], it ensures that the SIL isomerization is avoided, and confers chemoselectivity that is necessary for SIL which possesses multiple reactive moieties and also presents sensitivity to several reaction conditions [46]. SIL contains a total of five hydroxyl groups – three phenolic groups (5-OH, 7-OH, and 20-OH) and two alcoholic groups (3-OH and 23-OH) – which serve as the main targets for derivatization [46, 47]. However, the esterification of SIL using lipases selectively occurs in the 23-OH position of the flavolignan skeleton [46]. The production of SIL-O and SIL-L as monoesters was confirmed through vibrational spectroscopy, DFT analysis, and ^1^H liquid state NMR spectroscopy (Figures 1, 2, and 4, and Table 1), while the molecular structures of the obtained derivatives are presented in Figure 3. The synthesis of SIL derivatives using lipase-based conjugation with OA and LA was previously reported in a study aiming at the complexation of these esters with β-cyclodextrin [48]. Also, the biocatalytic modification of SIL using immobilized Candida antarctica lipase B was previously employed by Theodosiou et al. who resorted to this method for the obtainment of SIL lipophilic derivatives with dicarboxylic acids [49], as well as with FAs and their corresponding vinyl or methyl esters [50].

Next, a computational prediction of the properties (e.g., MW, cLogP, aqueous solubility, drug-likeness, drug score, and toxic risks) retained by SIL and the synthesized SIL-O and SIL-L derivatives was performed based on their chemical structures (Table 2). As expected, the complexation of SIL with UFAs resulted in compounds with higher MW and cLogP, as well as lower solubility. This observation correlates with previous reports suggesting that the conjugation of rutin, phloridzin, and esculin with FAs containing various chain lengths leads to derivatives with increased lipophilicity compared to the basic molecules [51]. SIL-O and SIL-L presented lower drug-likeness and drug score compared to SIL which could be explained by the increase in MW that is often associated with improved lipophilicity, but altered drug-like properties [52], and illustrates their applicability as prodrugs rather than active compounds. Although the drug score was identical for both derivatives, SIL-L presented a slightly higher drug-likeness compared to SIL-O. All investigated compounds were predicted to lack risk for mutagenic, tumorigenic, irritant, and reproductive toxicity.

The study further proceeded to an experimental investigation of SIL-O and SIL-L in terms of potential toxicity using in vitro biological models. The toxicological screening of the investigated compounds was performed using established cell lines –H9c2(2-1), HepaRG, and HaCaT – based on the data from the literature regarding their characteristics and applications as in vitro models. H9c2(2-1) are spindle-shaped cells, with similar morphology to embryonic cardiomyocytes. Their applications include cardiotoxicity testing and investigations regarding the cardioprotective effects retained by active compounds [25]. The HepaRG cell line, composed of two different populations of hepatic cells (cholangiocyte- and hepatocyte-like, respectively) are alternatives to primary human hepatocytes which can be employed as models in toxicity and metabolism studies [53]. HaCaT are nontumorigenic cells that present similarities to isolated keratinocytes in terms of morphology, major surface markers, and functional roles. They are also adapted for long-term culture and form a well-structured epidermis following in vivo transplantation [54]. The HaCaT cell line is commonly used as an in vitro model for toxicity assessment [25, 55]. The concentrations tested in this toxicological study were selected based on a review of previous publications assessing the impact of SIL on the viability of healthy cell lines [56–58].

The cytotoxic potential of SIL, SIL-O, and SIL-L at cardiac, hepatic, and cutaneous levels was assessed by exploring their impact on H9c2(2-1), HepaRG, and HaCaT cells’ viability following a 24 h treatment (Figure 5). Regarding the cytotoxicity of SIL in these healthy cell lines, a stimulatory effect on the viability of H9c2(2-1) cells at all tested concentrations was observed, while in HepaRG and HaCaT cells, SIL stimulated the viability at low concentrations and reduced it at high concentrations. These results reflecting that the cytotoxic effects of SIL depend on the tested concentration, cell type, and incubation period were previously reported in other studies. For instance, Anestopoulos et al. [59] showed that SIL was not cytotoxic to H9c2 cells at concentrations up to 200 µM after 48 h of treatment. In another study, SIL (60 and 90 µM) significantly decreased the viability of HK-2 human renal tubular endothelial cells after a 48 h treatment [60]. Gažák et al. [24] observed that SIL at a high concentration of 800 µM exerts no cytotoxic effect in Madin–Darby Canine Kidney (MDCK) cells after a treatment of 2 h.

As concerns the cytotoxic potential of the SIL derivatives, both SIL-O and SIL-L induced a higher decrease in cell viability compared to SIL, especially at the concentration of 100 µM. This observation is in concordance with a previous study revealing that the acyl-derivatives of SIL (obtained by the substitution of the 7-OH and 23-OH positions) exhibited higher cytotoxicity compared to the parent flavolignan SIL which increased with the acyl length. Notable cytotoxicity was recorded in the case of three SIL esters, namely 7-O-octanoyl, 23-O-octanoyl, and 23-O-dodecanoyl which might have resulted from their increased ability to cross cell membranes [24]. Similarly, Kubiak-Tomaszewska et al. demonstrated that the derivatization of flavonoids with FAs increases their cytotoxic activity. Specifically, they observed that the conjugates of 6-hydroxy-flavanone and 7-hydroxyflavone with stearic, oleic, sorbic, linolenic, and linoleic FAs exerted an enhanced cytotoxic potential toward HaCaT cells, presenting lower IC_50_ values compared to the respective flavonoids [61]. Comparatively, Warnakulasuriya et al. [62] found that the 48 h treatment with oleic, linoleic, and α-linolenic esters of quercetin-3-O-glucoside (at concentrations between 0.01 and 200 µM) lacked cytotoxicity in WI-38 fibroblasts, while a reduction in cell viability was obtained in the case of the highest concentrations (100 and 200 µM) of stearic, eicosapentaenoic acid, and docosahexaenoic acid derivatives. Another study unveiled that the presence of the lipid component influences the cytotoxicity of quercetin in ARPE-19 retinal pigment epithelial cells, which was dependent not only on the FA’s nature but also on the position at which the conjugation was performed. High toxicity was obtained in the case of the docosahexaenoic acid conjugate obtained through the substitution of the 3-OH of quercetin and was annulled when the same FA was introduced in the position 7-OH [63]. Recently, we have reported the biosynthesis and in vitro toxicological screening (on H9c2(2-1), HepaRG, and HaCaT cells) of two rutin oleate and linoleate conjugates (RUT-O and RUT-L) which were produced in the same manner as SIL derivatives [25]. To briefly compare the results, SIL-O behaved similarly to RUT-O (cell viabilities were around 70%–80% in all three cell types at 100 µM). However, contrary to the observations previously made in the case of RUT-L which showed increased biocompatibility in vitro on all tested cell lines and at all tested concentrations, the derivatization of SIL with LA resulted in a compound (SIL-L) with increased cytotoxicity at 100 µM. A strong correlation has been already found between the lipophilicity of flavonoid derivatives and their cytotoxic potential: linoleic flavone and flavanone derivatives were shown to have higher IC_50_ values in the healthy cell line HaCaT compared to oleic derivatives, and thus higher biocompatibility [51, 61] these data following what was observed in the case of the flavonoid rutin. However, in the case of the flavolignan SIL, an opposite outcome was obtained. SIL-L showed significantly higher cytotoxicity on H9c2(2-1), HepaRG, and HaCaT cells compared to SIL-O, despite its lower lipophilicity (as evidenced by the computational predictions made in Table 2). Despite causing a higher impairment in the viability of H9c2(2-1), HepaRG, and HaCaT cells compared to SIL, the obtained derivatives presented distinct cytotoxic profiles, especially at the highest concentration tested. According to the ISO Standard 10993-5:2009, a compound presents cytotoxicity if the cell viability following treatment reduces by at least 30% (or under 70%) [64]. Based on this estimation, SIL-O can be classified as non-cytotoxic on H9c2(2-1), HepaRG, and HaCaT cells, while only SIL-L showed cytotoxicity, the percentages of viable cells being lowered at values <60% at 100 µM.

Considering that morphological features play a central role in describing the underlying cell death mechanisms [65], the H9c2(2-1), HepaRG, and HaCaT cells’ morphology, confluence, and nuclear aspect (Figures 6–8) at the end of the 24-h treatment with SIL, SIL-O, and SIL-L 100 µM were further analyzed to explore whether these compounds exert an apoptotic or necrotic effect at this high concentration. During apoptosis, the organelles and cell membrane are conserved while the nucleus is early degenerated [66]. Other classical apoptotic features include cell shrinkage, membrane blebbing, nuclear condensation and fragmentation, chromatin cleavage, and formation of apoptotic bodies, characteristics that can be accurately identified through light and fluorescence microscopy [67, 68]. In necrosis, the nucleus remains intact, while the organelles and cell membrane undergo early degeneration [66]. Out of the tested compounds, SIL-L was found to produce the most significant apoptosis hallmarks in H9c2(2-1), HepaRG, and HaCaT cells at the concentration of 100 µM such as considerable morphological changes (e.g., shrinkage and rounding), accompanied by a substantial confluence loss, massive nuclear fragmentation, dysmorphology, and condensation, and increased AI. A preceding report by Nair et al. showed that the exposure of HepG2 cells to the derivatives of the flavonoid glucoside phloridzin, obtained through its regioselective acylation with a series of FAs (i.e., stearic acid ester, OA, LA, a-LA, docosahexaenoic acid, and eicosapentaenoic acid), at a concentration of 100 µM, caused cell detachment from the plate, accompanied by shrinkage, distortion of the membrane structure, and condensation of the nuclear chromatin, morphological features that are indicative of apoptotic cell death [69].

The last in vitro experiment assessed the influence of SIL, SIL-O, and SIL-L at low concentrations (1, 10, and 25 µM) on the migration of healthy H9c2(2-1), HepaRG, and HaCaT cells was investigated (Figure 9). The results were highly dependent on the tested compound, concentration, and cell type. Apart from HaCaT cells in which all compounds induced a similar effect on cell migration, SIL, SIL-O, and SIL-L caused different impacts on the migratory ability of cardiomyoblasts and hepatocytes. In H9c2(2-1) cells, SIL produced a dose-dependent stimulation of cell migration, while SIL-O and SIL-L showed an inhibitory effect at the highest concentration tested (25 µM). In HepaRG cells, SIL exerted an inhibitory effect on cell motility that was more prominent at 10 and 25 µM, while SIL-O only slightly reduced their migration, and SIL-L caused no impairments at the evaluated concentrations. A previous study reported the inhibitory effect of SIL on the migratory properties of LX-2 human hepatic stellate cells at concentrations of 10, 50, and 100 µM [70]. Comparatively to the results obtained for SIL-O and SIL-L, the conjugates synthesized through the derivatization of rutin with OA and LA at the concentration of 25 µM had no impact on the migration of H9c2(2-1) and HaCaT cells, while only inhibiting the migratory ability of HepaRG cells [25]. Carullo et al. [71] showed that the 24-h treatment of HaCaT cells with the hybrid molecule quercetin-3-oleate at concentrations of 0.1, 0.1, and 1 µM caused an enhancement in wound healing by 47%, 35%, and 51% compared to control.

The final interest of the present study was the in ovo evaluation of SIL-O and SIL-L (100 µM), in terms of vascular toxicity and irritant potential (Figure 11 and Table 3), by resorting to the HET-CAM assay which has been already accepted in many European countries as a full replacement method for the evaluation of severe irritants in animal models. The CAM is a vascularized tissue formed of arteries, capillaries, and veins that allows the differentiation between irritant and non-irritant substances through the evaluation of three vascular processes, namely hemorrhage, lysis, and coagulation, after the direct contact of the sample with the CAM [72]. SIL-O was classified as non-irritant, a result that is in accordance with the computational predictions (Table 2), inducing only minor signs of vascular lysis, while SIL-L exerted a slight irritant effect, causing microhemorrhage at the end of the exposure time. To the best of our knowledge, the irritant potential of SIL-O and SIL-L has not been investigated so far. However, we have previously reported the lack of irritant effect on the CAM and EpiDerm 3D microtissues of the FAs esters (at the concentration of 100 µM) obtained through the conjugation of RUT with OA and LA [25].

## Conclusion

To conclude, the conjugation of SIL with OA and LA led to the obtainment of two ester-type derivatives (SIL-O and SIL-L, respectively) that presented distinct safety profiles in vitro and in ovo. SIL-O lacked irritant potential and induced a lower toxicity compared to SIL-L which exerted cytotoxicity and a slight irritant effect at the highest concentration tested of 100 µM. Based on the findings presented herein, the biocompatibility of SIL-O and SIL-L as potential lipid-based prodrugs with applications in cardiac, hepatic, or skin injuries should be further addressed in in vivo studies.

## Supplemental data

Supplementary data are available at the following link: https://bjbms.org/ojs/index.php/bjbms/article/view/10600/3336

## Data Availability

The data are available at request from the corresponding author.

## References

[1] Sen T, Samanta SK (2014). Medicinal plants, human health and biodiversity: a broad review. Adv Biochem Eng Biotechnol.

[2] Falzon CC, Balabanova A (2017). Phytotherapy: an introduction to herbal medicine. Prim Care.

[3] Dehelean CA, Marcovici I, Soica C, Mioc M, Coricovac D, Iurciuc S (2021). Plant-derived anticancer compounds as new perspectives in drug discovery and alternative therapy. Molecules.

[4] Abushouk AI, Ismail A, Salem AMA, Afifi AM, Abdel-Daim MM (2017). Cardioprotective mechanisms of phytochemicals against doxorubicin-induced cardiotoxicity. Biomed Pharmacother.

[5] Shah SMA, Akram M, Riaz M, Munir N, Rasool G (2019). Cardioprotective potential of plant-derived molecules: a scientific and medicinal approach. Dose-Response.

[6] Mounika S, Jayaraman R, Jayashree D, Hanna Pravalika K, Balaji A, Banu MS (2021). A comprehensive review of medicinal plants for cardioprotective potential. Int J Adv Pharm Biotechnol.

[7] Tuli HS, Mittal S, Aggarwal D, Parashar G, Parashar NC, Upadhyay SK (2021). Path of Silibinin from diet to medicine: a dietary polyphenolic flavonoid having potential anti-cancer therapeutic significance. Semin Cancer Biol.

[8] Fanoudi S, Alavi MS, Karimi G, Hosseinzadeh H (2020). Milk thistle (Silybum Marianum) as an antidote or a protective agent against natural or chemical toxicities: a review. Drug Chem Toxicol.

[9] Bijak M (2017). Silybin, a major bioactive component of milk thistle (*Silybum Marianum* L. Gaernt.)—chemistry, bioavailability, and metabolism. Molecules.

[10] Loguercio C, Festi D (2011). Silybin and the liver: from basic research to clinical practice. World J Gastroenterol.

[11] Gillessen A, Schmidt HHJ (2020). Silymarin as supportive treatment in liver diseases: a narrative review. Adv Ther.

[12] Takke A, Shende P (2019). Nanotherapeutic silibinin: an insight of phytomedicine in healthcare reformation. Nanomedicine.

[13] Razavi BM, Karimi G (2016). Protective effect of silymarin against chemical-induced cardiotoxicity. Iran J Basic Med Sci.

[14] Chen YH, Lin H, Wang Q, Hou JW, Mao ZJ, Li YG (2020). Protective role of silibinin against myocardial ischemia/reperfusion injury-induced cardiac dysfunction. Int J Biol Sci.

[15] Singh RP, Agarwal R (2009). Cosmeceuticals and silibinin. Clin Dermatol.

[16] Liu W, Wang F, Li C, Otkur W, Hayashi T, Mizuno K (2021). Silibinin treatment protects human skin cells from UVB injury through upregulation of estrogen receptors. J Photochem Photobiol B.

[17] Kaur M, Deep G, Agarwal R.

[18] Theodosiou E, Purchartová K, Stamatis H, Kolisis F, Křen V (2014). Bioavailability of silymarin flavonolignans: drug formulations and biotransformation. Phytochem Rev.

[19] Tvrdý V, Pourová J, Jirkovský E, Křen V, Valentová K, Mladěnka P (2021). Systematic review of pharmacokinetics and potential pharmacokinetic interactions of flavonolignans from silymarin. Med Res Rev.

[20] Tang N, Wu D, Lu Y, Chen J, Zhang B, Wu W (2009). A comparative study on the stability of silybin and that in silymarin in buffers and biological fluids. Drug Metab Lett.

[21] Markovic M, Ben-Shabat S, Dahan A (2020). Prodrugs for improved drug delivery: lessons learned from recently developed and marketed products. Pharmaceutics.

[22] Han S, Mei L, Quach T, Porter C, Trevaskis N (2021). Lipophilic conjugates of drugs: a tool to improve drug pharmacokinetic and therapeutic profiles. Pharm Res.

[23] Fattahi N, Shahbazi MA, Maleki A, Hamidi M, Ramazani A, Santos HA (2020). Emerging insights on drug delivery by fatty acid mediated synthesis of lipophilic prodrugs as novel nanomedicines. J Control Release.

[24] Gažák R, Purchartová K, Marhol P, živná L, Sedmera P, Valentová K (2010). Antioxidant and antiviral activities of silybin fatty acid conjugates. Eur J Med Chem.

[25] Dehelean CA, Coricovac D, Pinzaru I, Marcovici I, Macasoi IG, Semenescu A (2022). Rutin bioconjugates as potential nutraceutical prodrugs: an in vitro and in ovo toxicological screening. Front Pharmacol.

[26] Bhat M (2021). Opportunities and challenges of fatty acid conjugated therapeutics. Chem Phys Lipids.

[27] Wang W, Chen K, Xia Y, Mo W, Wang F, Dai W (2018). The hepatoprotection by oleanolic acid preconditioning: focusing on PPAR*α* activation. PPAR Res.

[28] Cardoso CR, Favoreto S, Oliveira LL, Vancim JO, Barban GB, Ferraz DB (2011). Oleic acid modulation of the immune response in wound healing: a new approach for skin repair. Immunobiology.

[29] Kendall AC, Kiezel-Tsugunova M, Brownbridge LC, Harwood JL, Nicolaou A (2017). Lipid functions in skin: differential effects of n-3 polyunsaturated fatty acids on cutaneous ceramides, in a human skin organ culture model. Biochim Biophys Acta.

[30] Kerrhard AL, Pegg RB, Sarkar A, Craft BD (2015). Update on the methods for monitoring UFA oxidation in food products. Eur J Lipid Sci Technol.

[31] Frisch MJ, Trucks GW, Schlegel HB, Scuseria GE, Robb MA, Cheeseman JR, et al.

[32] Bannwarth C, Ehlert S, Grimme S (2019). GFN2-xTB—an accurate and broadly parametrized self-consistent tight-binding quantum chemical method with multipole electrostatics and density-dependent dispersion contributions. J Chem Theory Comput.

[33] Pracht P, Bohle F, Grimme S (2020). Automated exploration of the low-energy chemical space with fast quantum chemical methods. Phys Chem Chem Phys.

[34] Grimme S (2019). Exploration of chemical compound, conformer, and reaction space with meta-dynamics simulations based on tight-binding quantum chemical calculations. J Chem Theory Comput.

[35] Pracht P, Grimme S.

[36] Ayati A, Falahati M, Irannejad H, Emami S (2012). Synthesis, in vitro antifungal evaluation and in silico study of 3-azolyl-4-chromanone phenylhydrazones. Daru.

[37] Gag O, Macasoi I, Pinzaru I, Dinu S, Popovici R, Cosoroaba MR (2023). In vitro assessment of the impact of ultraviolet B radiation on oral healthy and tumor cells. Photonics.

[38] Pinzaru I, Chioibas R, Marcovici I, Coricovac D, Susan R, Predut D (2021). Rutin exerts cytotoxic and senescence-inducing properties in human melanoma cells. Toxics.

[39] Breban-Schwarzkopf D, Chioibas R, Macasoi I, Bolintineanu S, Marcovici I, Draghici G (2024). Comprehensive in vitro and in ovo assessment of cytotoxicity: Unraveling the impact of sodium fluoride, xylitol, and their synergistic associations in dental products. Biomol Biomed Online ahead of print..

[40] Sahu RK, Singh B, Saraf SA, Kaithwas G, Kishor K (2014). Photochemical toxicity of drugs intended for ocular use. Arh Hig Rada Toksikol.

[41] Qiu J, Hou H-Y, Huyen NT, Yang I-S, Chen X-B (2019). Raman spectroscopy and 2DCOS analysis of unsaturated fatty acid in edible vegetable oils. Appl Sci.

[42] Machado NFL, De Carvalho LAEB, Otero JC, Marques MPM (2012). The autooxidation process in linoleic acid screened by Raman spectroscopy. J Raman Spectrosc.

[43] Gažák R, Marhol P, Purchartová K, Monti D, Biedermann D, Riva S (2010). Large-scale separation of silybin diastereoisomers using lipases. Process Biochem.

[44] Kadoglou NPE, Panayiotou C, Vardas M, Balaskas N, Kostomitsopoulos NG, Tsaroucha AK (2022). A comprehensive review of the cardiovascular protective properties of silibinin/silymarin: a new kid on the block. Pharmaceuticals.

[45] Ortiz C, Ferreira ML, Barbosa O, Dos Santos JCS, Rodrigues RC, Berenguer-Murcia Á (2019). Novozym 435: the “perfect” lipase immobilized biocatalyst?. Catal Sci Technol.

[46] Biedermann D, Vavříková E, Cvak L, Křen V (2014). Chemistry of silybin. Nat Prod Rep.

[47] Fallah M, Davoodvandi A, Nikmanzar S, Aghili S, Mirazimi SMA, Aschner M (2021). Silymarin (milk thistle extract) as a therapeutic agent in gastrointestinal cancer. Biomed Pharmacother.

[48] Pînzaru IA, Hădărugă DI, Hădărugă NG, Corpaş L, Grozescu I, Peter F (2011). Hepatoprotective flavonoid bioconjugate/β-cyclodextri nanoparticles: DSC—molecular modeling correlation. Dig J Nanomater Biostruct.

[49] Theodosiou E, Loutrari H, Stamatis H, Roussos C, Kolisis FN (2011). Biocatalytic synthesis and antitumor activities of novel silybin acylated derivatives with dicarboxylic acids. New Biotechnol.

[50] Theodosiou E, Katsoura MM, Loutrari H, Purchartová K, Křen V, Kolisis FN (2009). Enzymatic preparation of acylated derivatives of silybin in organic and ionic liquid media and evaluation of their antitumor proliferative activity. Biocatal Biotransform.

[51] Danihelová M, Viskupičová J, Šturdík E (2012). Lipophilization of flavonoids for their food, therapeutic and cosmetic applications. Acta Chim Slovaca.

[52] Naylor MR, Ly AM, Handford MJ, Ramos DP, Pye CR, Furukawa A (2018). Lipophilic permeability efficiency reconciles the opposing roles of lipophilicity in membrane permeability and aqueous solubility. J Med Chem.

[53] Mann A, Pelz T, Rennert K, Mosig A, Decker M, Lupp A (2017). Evaluation of HepaRG cells for the assessment of indirect drug-induced hepatotoxicity using INH as a model substance. Hum Cell.

[54] Colombo I, Sangiovanni E, Maggio R, Mattozzi C, Zava S, Corbett Y (2017). HaCaT cells as a reliable in vitro differentiation model to dissect the inflammatory/repair response of human keratinocytes. Mediat Inflamm.

[55] Ridd K, Dhir S, Smith AG, Gant TW (2010). Defective TPA signalling compromises HaCat cells as a human in vitro skin carcinogenesis model. Toxicol In Vitro.

[56] Song XY, Li RH, Liu WW, Mizuno K, Hattori S, Fujisaki H (2021). Effect of silibinin on ethanol- or acetaldehyde-induced damage of mouse primary hepatocytes in vitro. Toxicol Vitro.

[57] Goh ZH, Tee JK, Ho HK (2020). An evaluation of the in vitro roles and mechanisms of silibinin in reducing pyrazinamide-and isoniazid-induced hepatocellular damage. Int J Mol Sci.

[58] Ma Z, Zang W, Wang H, Wei X (2020). Silibinin enhances anti-renal fibrosis effect of MK-521 via downregulation of TGF-β signaling pathway. Hum Cell.

[59] Anestopoulos I, Kavo A, Tentes I, Kortsaris A, Panayiotidis M, Lazou A (2013). Silibinin protects H9c2 cardiac cells from oxidative stress and inhibits phenylephrine-induced hypertrophy: potential mechanisms. J Nutr Biochem.

[60] Liu R, Wang Q, Ding Z, Zhang X, Li Y, Zang Y (2020). Silibinin augments the antifibrotic effect of valsartan through inactivation of TGF-β1 signaling in kidney. Drug Des Devel Ther.

[61] Kubiak-Tomaszewska G, Roszkowski P, Grosicka-Maciag E, Strzyga-Łach P, Struga M (2022). Effect of hydroxyl groups esterification with fatty acids on the cytotoxicity and antioxidant activity of flavones. Molecules.

[62] Warnakulasuriya SN, Ziaullah, Rupasinghe HP (2016). Long chain fatty acid esters of quercetin-3-O-glucoside attenuate H_2_O_2_-induced acute cytotoxicity in human lung fibroblasts and primary hepatocytes. Molecules.

[63] Moine E, Brabet P, Guillou L, Durand T, Vercauteren J, Crauste C (2018). New lipophenol antioxidants reduce oxidative damage in retina pigment epithelial cells. Antioxidants.

[64] Racea RC, Macasoi IG, Dinu S, Pinzaru I, Marcovici I, Dehelean C (2023). Eugenol: in vitro and in ovo assessment to explore cytotoxic effects on osteosarcoma and oropharyngeal cancer cells. Plants.

[65] Ziegler U, Groscurth P (2004). Morphological features of cell death. News Physiol Sci.

[66] Eidet JR, Pasovic L, Maria R, Jackson CJ, Utheim TP (2014). Objective assessment of changes in nuclear morphology and cell distribution following induction of apoptosis. Diagn Pathol.

[67] Doonan F, Cotter TG (2008). Morphological assessment of apoptosis. Methods.

[68] Surducan DA, Racea RC, Cabuta M, Olariu I, Macasoi I, Rusu LC (2023). Eugenol induces apoptosis in tongue squamous carcinoma cells by mediating the expression of Bcl-2 family. Life.

[69] Nair SV, Ziaullah, Rupasinghe HP (2014). Fatty acid esters of phloridzin induce apoptosis of human liver cancer cells through altered gene expression. PLoS One.

[70] Ezhilarasan D, Evraerts J, Brice S, Buc-Calderon P, Karthikeyan S, Sokal E (2016). Silibinin inhibits proliferation and migration of human hepatic stellate LX-2 cells. J Clin Exp Hepatol.

[71] Carullo G, Governa P, Leo A, Gallelli L, Citraro R, Cione E (2019). Quercetin-3-oleate contributes to skin wound healing targeting FFA1/GPR40. ChemistrySelect.

[72] Budai P, Kormos É, Buda I, Somody G, Lehel J (2021). Comparative evaluation of HET-CAM and ICE methods for objective assessment of ocular irritation caused by selected pesticide products. Toxicol Vitro.

