# Correction: Synthesis, characterization, and *in vitro*ー*in ovo* toxicological screening of silibinin fatty acids conjugates as prodrugs with potential biomedical applications

**DOI:** 10.17305/bb.2025.12570

**Published:** 2025-04-25



Cristina Dehelean, Ersilia Alexa, Iasmina Marcovici, Andrada Iftode, Geza Lazar, Andrea Simion, Vasile Chis, Adrian Pirnau, Simona Cinta Pinzaru, Estera Boeriu

Vol. 24 No. 6 (2024) | https://doi.org/10.17305/bb.2024.10600 | 17 October, 2024

The affiliation of the first author, **Cristina Adriana Dehelean**, was incomplete in the originally published version of this article. One of the authors asked to add her third institutional affiliation.

The correct affiliations for Cristina Adriana Dehelean are as follows:

^1^Faculty of Pharmacy, “Victor Babeș” University of Medicine and Pharmacy Timișoara, Timișoara, Romania

^2^Research Center for Pharmaco-Toxicological Evaluations, Faculty of Pharmacy, “Victor Babeș” University of Medicine and Pharmacy Timișoara, Timișoara, Romania

**^3^Faculty of Food Engineering, University of Life Sciences “King Michael I” from Timișoara, Timișoara, Romania.**

We apologize to the readership for any inconvenience caused.

